# Supplementary material for: Evolutionary conserved NSL complex/BRD4 axis controls transcription activation via histone acetylation
Source: Nat Commun. 2020 May 7;11:2243. doi: 10.1038/s41467-020-16103-0 (PMC7206058; doi:10.1038/s41467-020-16103-0)
Supplement: Supplementary file 1 — Supplementary Information [file 41467_2020_16103_MOESM1_ESM.pdf]

SUPPLEMENTARY INFORMATION

Evolutionary conserved NSL complex/BRD4 axis controls transcription activation via histone acetylation

Gaub *et al.*

Supplementary Figure 1

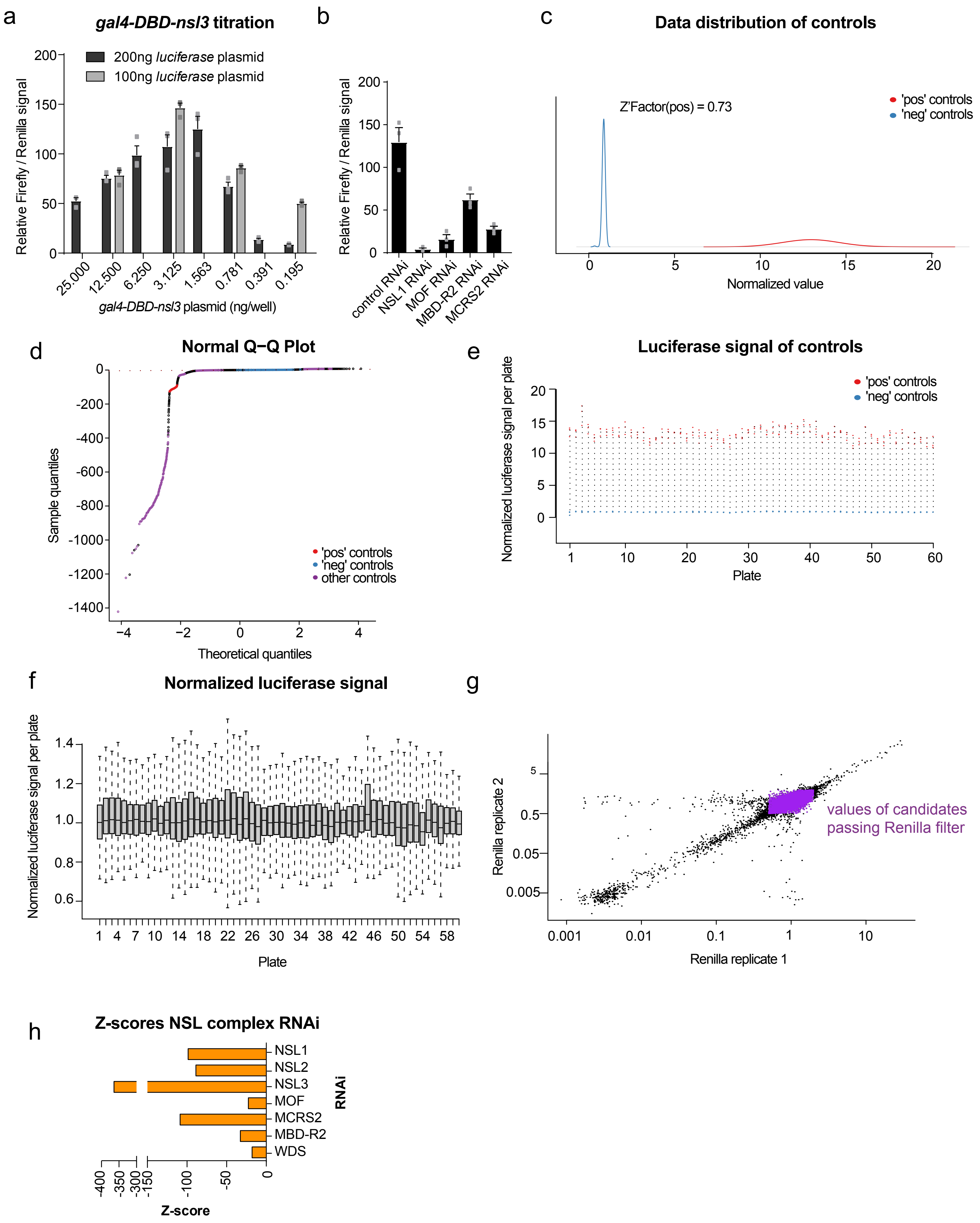

Supplementary Figure 1. Assay optimization and quality controls of genome-wide screen

a. Firefly luciferase signal normalized to the Renilla signal of each well. Titration of *Gal4-DBD-nsl3* "activator" plasmid (see Fig. 1). Data are presented as mean value ± SEM of n=3 technical replicates.

b. Firefly luciferase signal normalized over Renilla signal per well after RNAi of NSL complex members. Data are presented as mean value ± SEM of n=3 technical replicates.

c.-f. Quality control plots of genome wide RNAi screen using cell HTS2 analysis software. Luciferase data was plate median normalized (see methods for more details). For simplicity in **c., e., f.** only data plots of replicate 1 are shown.

c. Distribution of luciferase signal of the positive (red line, GST) and negative (blue line, NSL1) controls, obtained from kernel density estimates.

d. Normal Quantile-Quantile plot (QQ plot). Negative control (GST) in blue, positive control (NSL1) in red, other controls used (from left to right in QQ plot: NSL3, MOF, GFP, Diap1) in purple.

e. Normalized luciferase signal of positive (red, GST) and negative (blue, NSL1) controls per 384 well plate.

f. Boxplots of normalized luciferase signal intensities per plate. Boxplots represent median (centre), interquartile-range (bounds of box) and minima/maxima (whiskers) of data distributions.

g. Scatterplot of plate mean normalized Renilla reads of the first versus the second replicate. In black: candidates with normalized Renilla reads greater 2 or smaller than 0.5, which were removed from the candidate list for secondary assays. In purple: all candidates passing the Renilla filtering step.

h. Barplot represents z-scores of NSL complex members as obtained from the genome-wide RNAi screen.

Source data for **a**, **b** are provided as a Source Data file.

Supplementary Figure 2

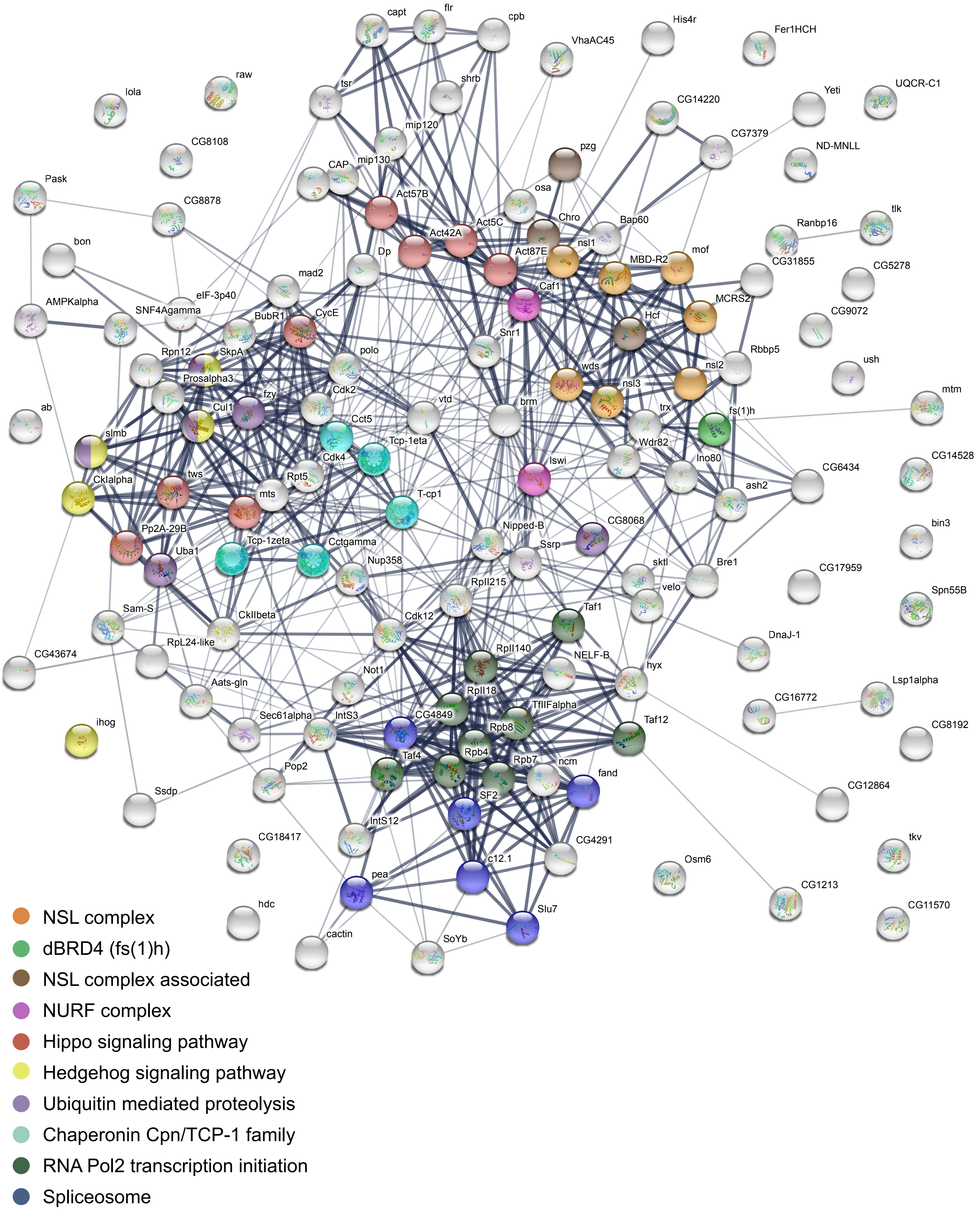

Supplementary Figure 2. Functional interactome of the NSL complex

Candidates passing primary and secondary screen criteria (> 1.5 fold activation in *Gal4-DBD-nsl3* based assay compared to full length *Gal4* assay in secondary screen). Interaction network was generated using the Search Tool for the Retrieval of Interacting Genes/Proteins (STRING) database of physical and functional interactions [1]. The thickness of the network edges indicates the interaction confidence.

Supplementary Figure 3

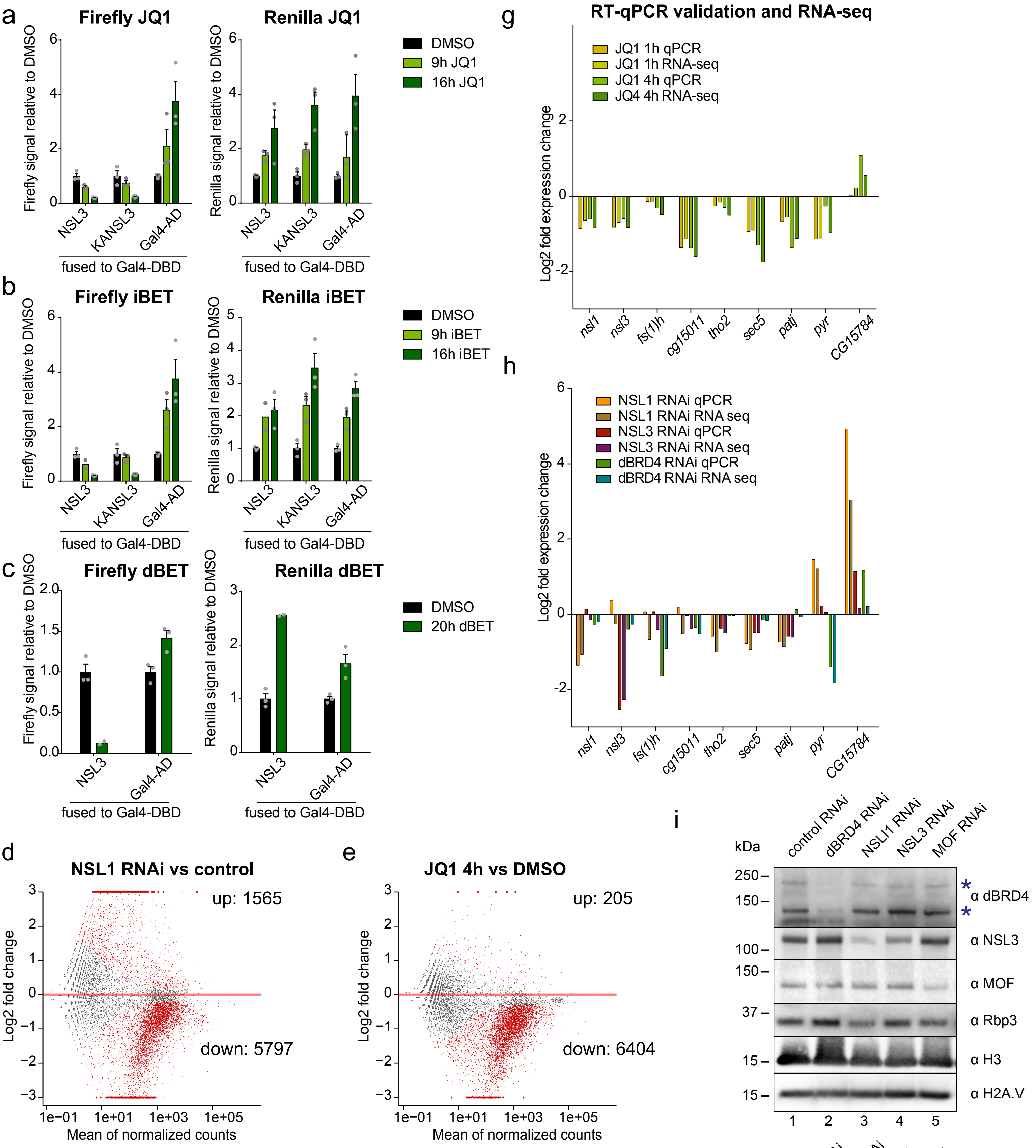

f **Welch two sample t-test: RNAi gene expression vs control**

	RNAi / number of genes	NSL1 RNAi	dBRD4 RNAi	dBRD4-L RNAi
NSL-bound	n=6000	1.2e-7	6.0 e-4	7.4 e-4
NSL-bound	n=2288	0.0015	0.035	0.042
NSL non-bound	n=2288	0.78	0.78	0.89

j **Immunoblot quantification**

	control RNAi	NSL1 RNAi	DMSO	1h JQ1	1h dBET	4h JQ1	4h dBET
Pol2 ser2p	1	0.59	1	0.56	0.57	0.68	0.66
Pol2 ser5p	1	0.62	1	0.88	1.04	0.81	0.93
Rpb3	1	0.66	1	0.87	0.86	0.79	0.82
Actin	1	1.24	1	0.98	1.03	1.23	0.94

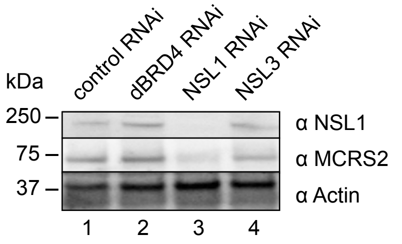

Supplementary Figure 3. Gene expression analysis following dBRD4 or NSL complex depletion

a-c. Firefly (as in Fig. 3b-d) and Renilla luciferase activity assayed using *Gal4-DBD-nsl3*, *Gal4-DBD-KANSL3* or full length *Gal4* driving the expression of the *UAS-firefly* reporter after DMSO or: **a)** 5µM JQ1, **b)** 1µM iBET 762, **c)** 100nM dBET6 inhibitor treatments. Data are presented as mean value ± SEM of n=3 technical replicates, except *Gal4-DBD-nsl3* activity after 9h dBET treatment in **b)** n=2 technical replicates. Source data are provided as a Source Data file.

d., e. MA plots of RNA-seq data, based on DESeq2 analysis. Red colour highlights all genes showing a significant differential expression (adjusted P value < 0.05). **d.** NSL1 RNAi versus control RNAi (GST), **e.** 4h JQ1 (5µM) treatment versus DMSO. Expression was normalized using synthetic ERCC spikes (see methods section for details).

f. Table listing *p*-values of Two-sided Welch Two Sample t-tests. Tests were performed on normalized RNA-seq counts of control RNAi vs NSL, dBRD4 and dBRD4-L RNAi for subsets of NSL complex-bound or unbound genes (see also Fig. 3f).

g., h. RT-qPCR validation of RNA-seq after **g.** 1h and 4h JQ1 treatment and **h.** NSL1, NSL3 and dBRD4 RNAi. RT-qPCRs were normalized to *ATPsynbeta*, *sun* and *SP1029* and are presented relative to DMSO or control RNAi, n=1 independent biological replicate for RT-qPCR validation.

i. Representative western blot of nuclear protein extracts showing RNAi efficiencies and effects on protein levels of NSL complex members and dBRD4 upon RNAi. H2A.V, H3, Rpb3 and Actin serving as loading control. The same extracts were loaded for both subpanels in this figure. Blue asterisks indicate long and short isoform of dBRD4. This experiment was repeated independently one more time showing similar results.

j. Relative quantifications of immunoblots, see also Fig. 3h,i. Numbers represent averages of two biological replicates.

Supplementary Figure 4

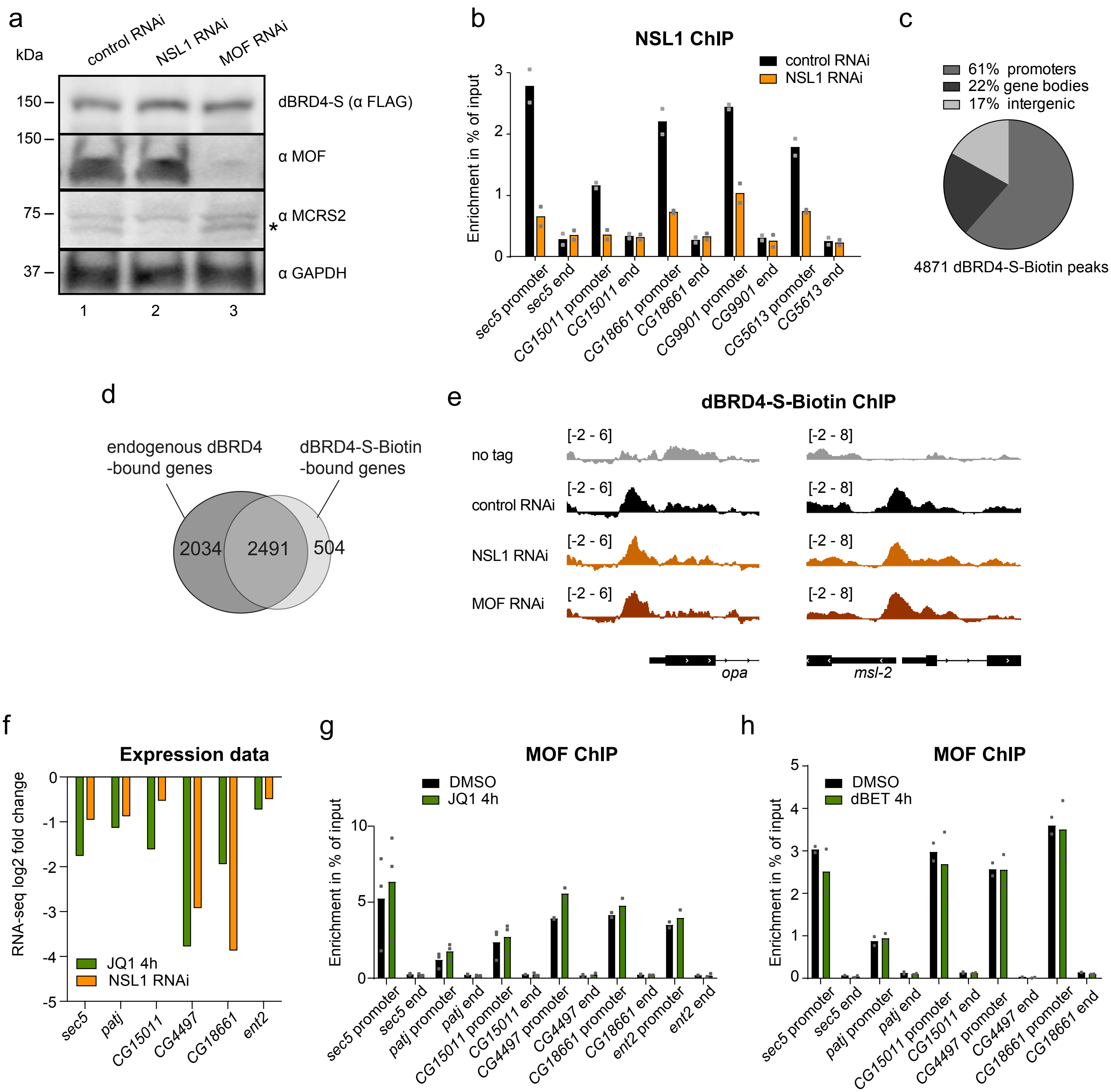

Supplementary Figure 4. dBET and NSL complex ChIP analyses

a. Representative western blot of nuclear extracts of control (GST) (lane 1), NSL1 (lane 2), and MOF (lane 3) RNAi in dBRD4-S-FLAG-Biotin overexpressing S2 cells. For NSL1 RNAi efficiency see panel **b.** RNAi efficiencies were tested for each RNAi experiment.

b. NSL1 ChIP-qPCR of control (GST) RNAi and NSL1 RNAi. Primers target the promoters and ends of NSL complex-bound genes.

c. Piechart on distribution of dBRD4-S-Biotin ChIP peaks falling onto promoters (TSS +/-200bp), gene bodies and intergenic regions.

d. Venn diagram of overlap of genes bound by endogenous dBRD4 antibody ChIP [2] and dBRD4-S-Biotin ChIP. Genes were considered bound if peaks called with MACS overlapped with +/-200bp from the annotated TSS.

e. IGV browser snapshots of dBRD4-S-Biotin ChIP-seq profiles in control (GST), NSL1 and MOF RNAi as well as in WT untagged cells. Input-subtracted ChIP-seq data (merge of two biological replicates) is shown (as in Fig. 4c).

f. Expression changes after 4h of JQ1 treatment (5µM) for genes assayed by NSL1 and MOF ChIP-qPCR (see Fig. 4e-g.) Expression changes plotted are from RNA-seq dataset (see Fig. 3e).

g. and **h.** MOF ChIP-qPCR after **g.** JQ1 (5µM) or **h.** dBET6 (100nM) or DMSO treatments for 4h. Primers target the promoters and ends of NSL complex and dBRD4-bound genes, for expression changes after 4h JQ1 treatment of these genes, see **f.**

For **b.**, **g.**, **h**.: Data are presented as mean value of n=2 or n=3 biological replicates. Source data are provided as a Source Data file.

Supplementary Figure 5

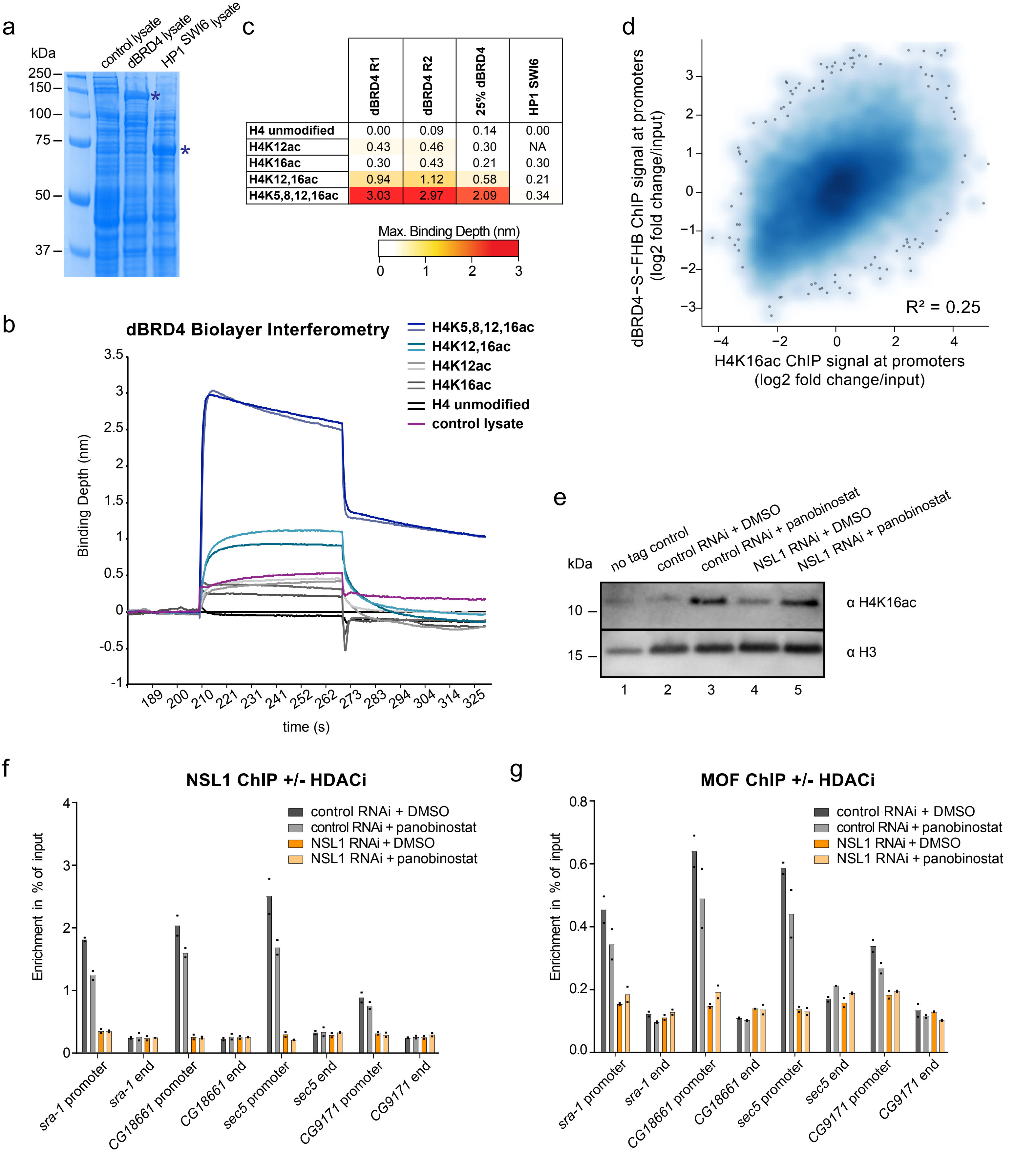

	dBRD4 R1	dBRD4 R2	25% dBRD4	HP1 SWI6
H4 unmodified	0.00	0.09	0.14	0.00
H4K12ac	0.43	0.46	0.30	NA
H4K16ac	0.30	0.43	0.21	0.30
H4K12,16ac	0.94	1.12	0.58	0.21
H4K5,8,12,16ac	3.03	2.97	2.09	0.34

Supplementary Figure 5. NSL mediated acetylation contributes to acH4 tail recognition of dBRD4

a. Representative coomassie-stained SDS-PAGE of bacteria lysates for biolayer interferometry experiments. Asterisks indicate expected molecular weight of SMT3-dBRD4-S and GST-HP1^{SWI6} respectively. This experiment was repeated independently three more times showing similar results.

b. Biolayer interferometry (BLItz) sensograms represent qualitative binding measurements of SMT3-dBRD4-S lysates and control lysates (obtained from uninduced bacteria) to modified H4 peptides (aminoacids 1-23). Data was standardized to binding signal to unmodified H4 peptides. Two technical replicates are shown, the experiment was validated with independent biological replicates (data not shown).

c. Values of maximum binding depth of biolayer interferometry measurements are given. 25% dBRD4 lysate was obtained by dilution with control lysate.

d. Scatterplot of dBRD4-S-Biotin ChIP signal and H4K16ac ChIP signal on gene promoters, signal for all gene promoters is plotted. (TSS +/-200bp for dBRD4-S-Biotin ChIP, TSS +500bp for H4K16ac ChIP). ChIP signals were normalized log2 fold change over input. R^2 was calculated with linear regression model.

e. Representative western blot of H4K16ac after panobinostat treatment (4h, 200nM) in control RNAi (GST, lanes 2, 3) or NSL1 RNAi (lanes 4, 5) cells. This experiment was repeated independently one more time showing similar results.

f., **g.** ChIP-qPCR of **f.** NSL1 and **g.** MOF after DMSO or HDAC inhibitor panobinostat treatment (4h, 200nM) in control RNAi (GST) or NSL1 RNAi cells. Primers target the promoters and ends of dBRD4 and NSL complex-bound genes. Data are presented as mean value of n=2 biological replicates. Source data are provided as a Source Data file.

Supplementary Figure 6

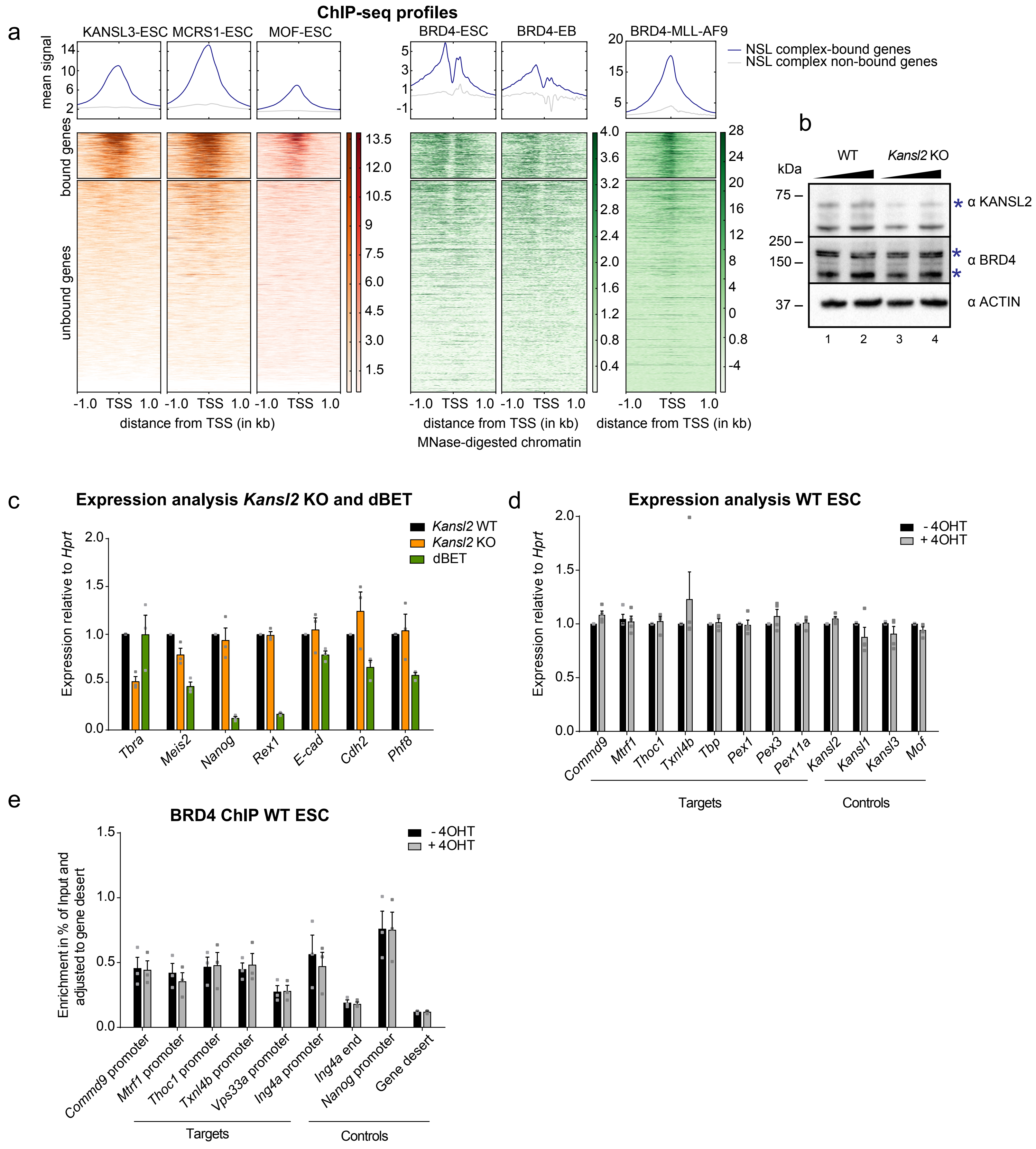

Supplementary Figure 6. Characterization of *Kansl2* KO mESC

a. Heatmaps and profile plots of publicly available ChIP-seq data of KANSL3, MCRS11, MOF [3] and BRD4 in mouse embryonic stem cells (mESC), BRD4 in mouse embryoid bodies (EB) [4] and BRD4 in MLL-AF9 cells [5]. BRD4 profiles from mESC and EB were generated by MNase ChIP-seq. All genes are plotted, clustered into two groups, clustering by k-means based on KANSL3 ChIP profile. Order of genes was maintained for all heatmaps.

b. Representative western blot of whole cell extracts of *Kansl2* p/p, Cre-ERT2 mESCs with (*Kansl2* KO) and without (*Kansl2* WT) tamoxifen treatment (3 days 500nM). Blue asterisks indicate protein specific bands (KANSL2, short and long isoform of BRD4 respectively). This experiment was repeated independently one more time showing similar results.

c. RT-qPCR analysis of *Kansl2* p/p, Cre-ERT2 mESCs with (*Kansl2* KO) and without (*Kansl2* WT) tamoxifen treatment (500nM, 3days), as well as *Kansl2* p/p, Cre-ERT2 cells cultured without tamoxifen, treated with dBET6 (100nM, 4h). See also Fig. 6a.

d. RT-qPCR analysis of wildtype Black 6 mESCs with and without tamoxifen treatment (500nM, 3 days).

e. ChIP-qPCR of BRD4 in wildtype Black 6 mESCs with and without tamoxifen treatment (500nM, 3 days).

For **c.-e.** Data are presented as mean value ± SEM of n=3 biological replicates. Source data are provided as a Source Data file.

Supplementary Figure 7

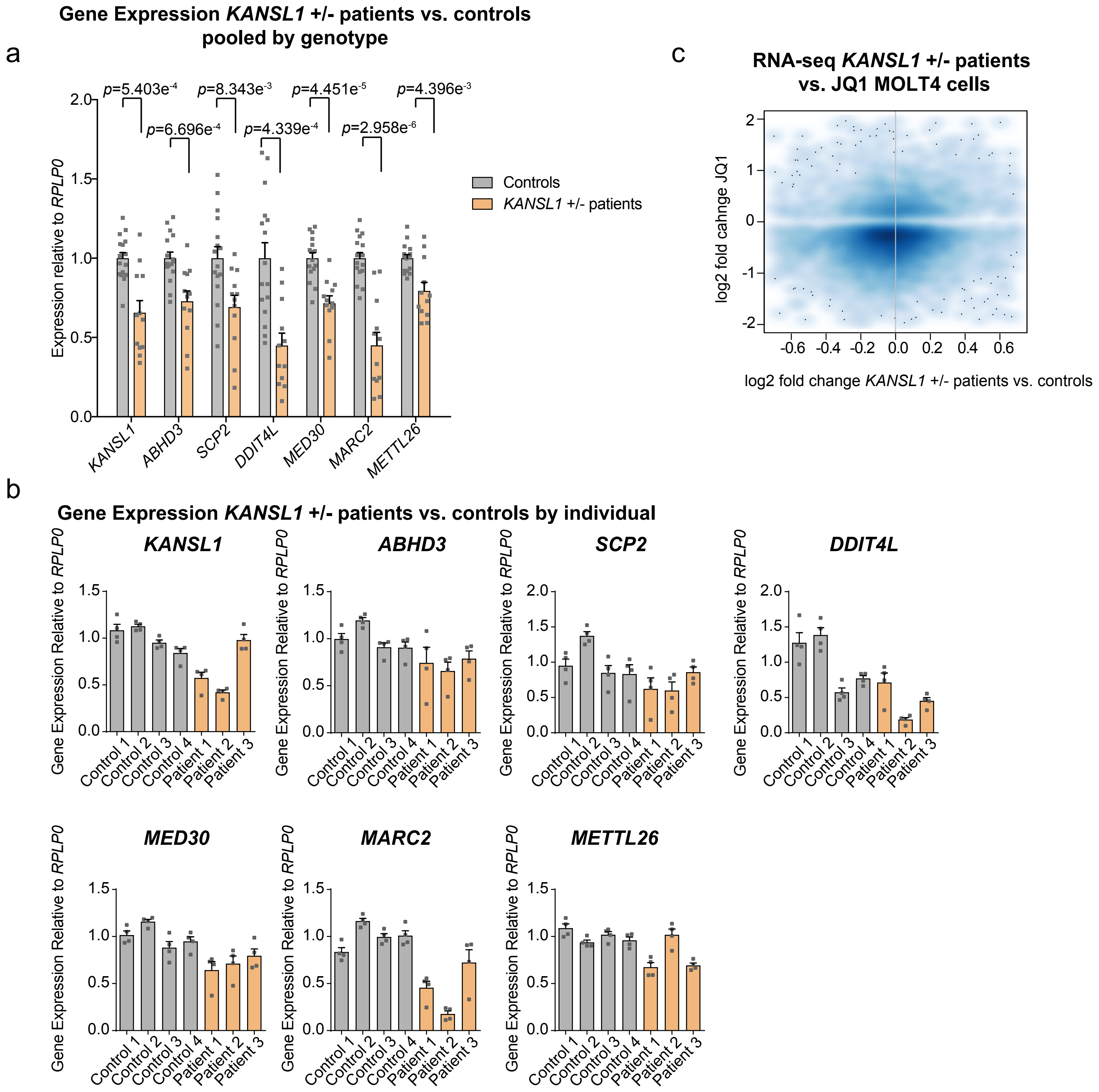

Supplementary Figure 7. Characterization KANSL1 +/- and control fibroblast cell lines

a. RT-qPCR validation of RNA-seq data from fibroblast cell lines of three Koolen-de Vries syndrome (*KANSL1* +/-) patients and four controls (see also Fig. 6c, d). Four biological replicates were analysed per cell line and the measurements pooled according to genotype (*KANSL1* +/- patient or control) Error bars represent SEM (n=12 for patients, n=16 for controls). Two-sided Wilcoxon Rank-Sum tests were applied to test significance levels.

b. As in **a**, but measurements are categorized according to individual cell lines derived from individual *KANSL1* +/- patients or controls. Error bars represent SEM of four biological replicates.

c. Scatterplot of log2 fold changes of gene expression from JQ1 (1µM, 2h) versus DMSO treated MOLT4 cells [6] and log2 fold changes of gene expression from *KANSL1* heterozygous patient fibroblasts versus control fibroblasts.

Source data for **a**, **b** are provided as a Source Data file.

Supplementary Figure 8

a. related to Figure 3a, i

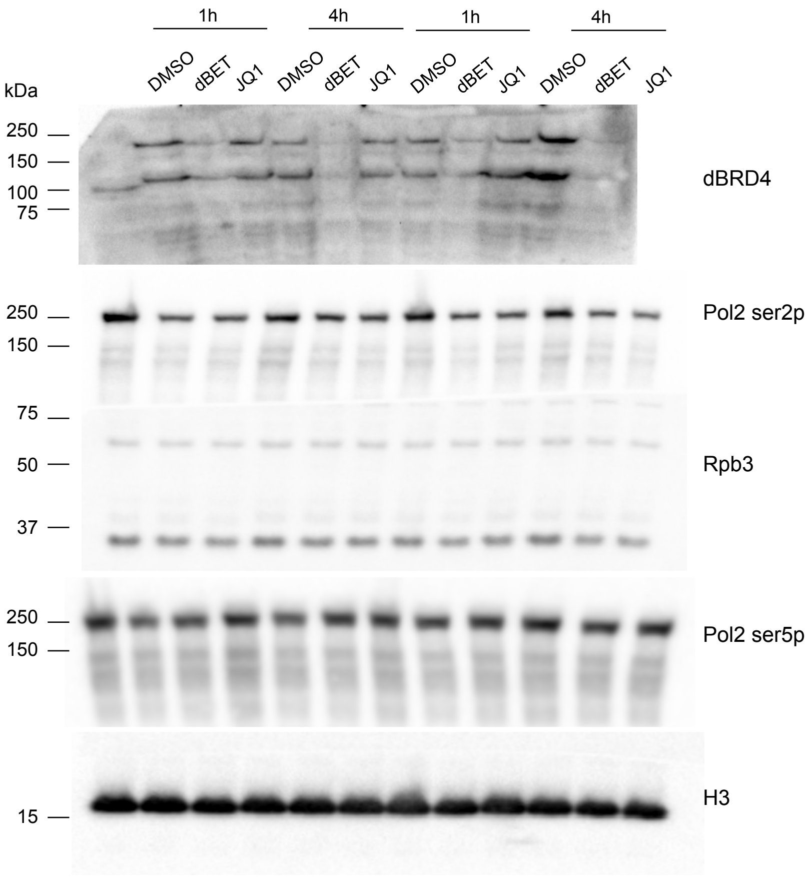

b. related to Figure 3h

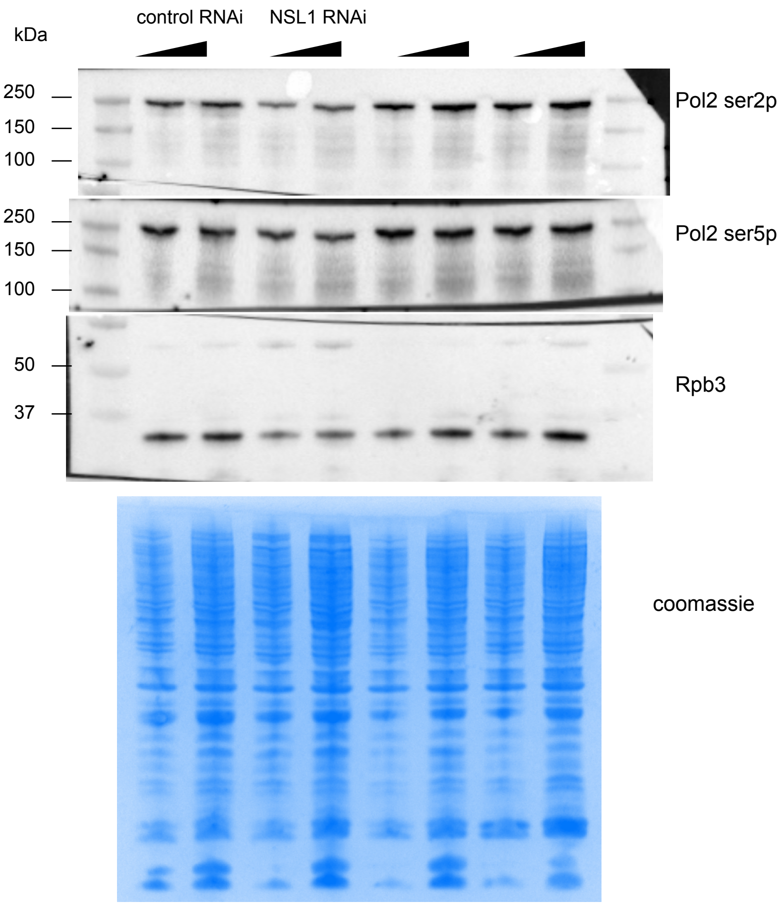

c. related to Supplementary Figure 3i, j

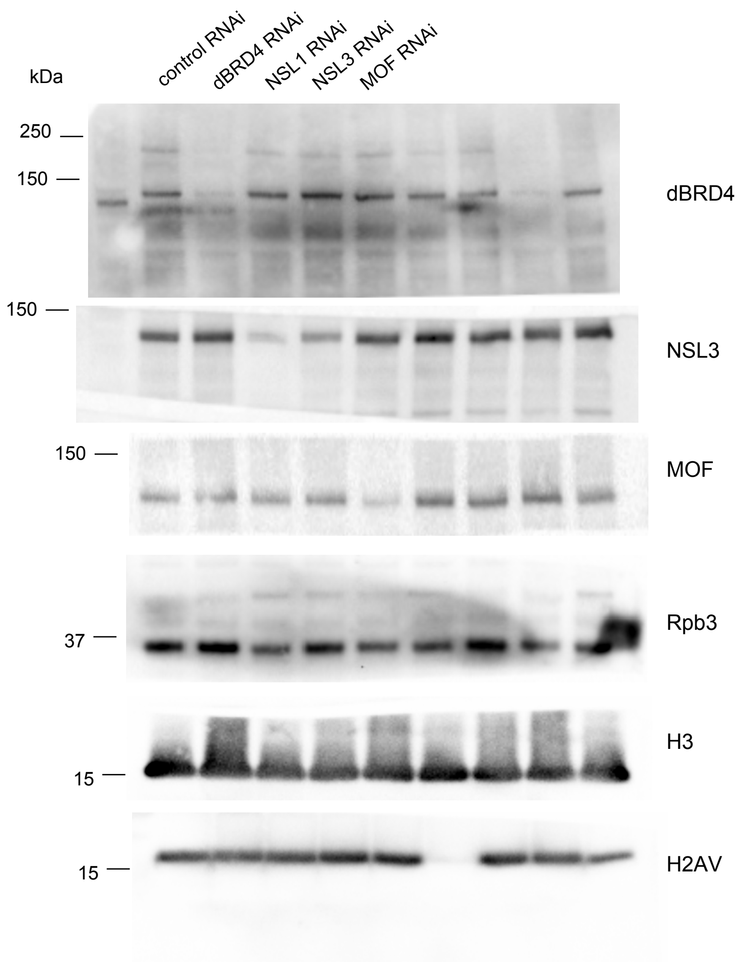

d. related to Supplementary Figure 3i, j

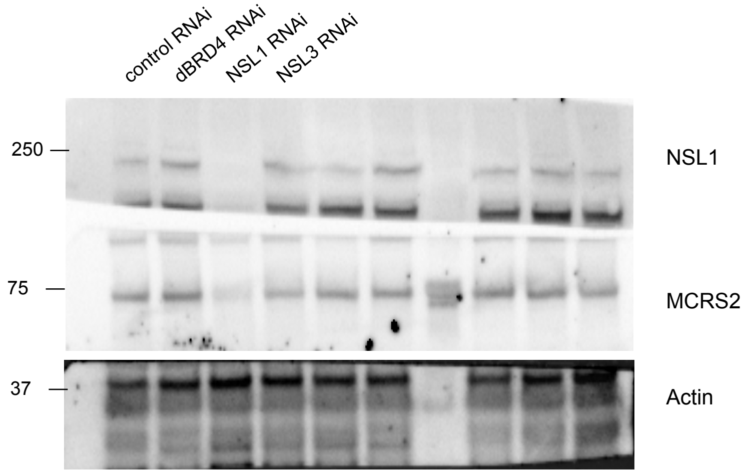

e. related to Supplementary Figure 4a

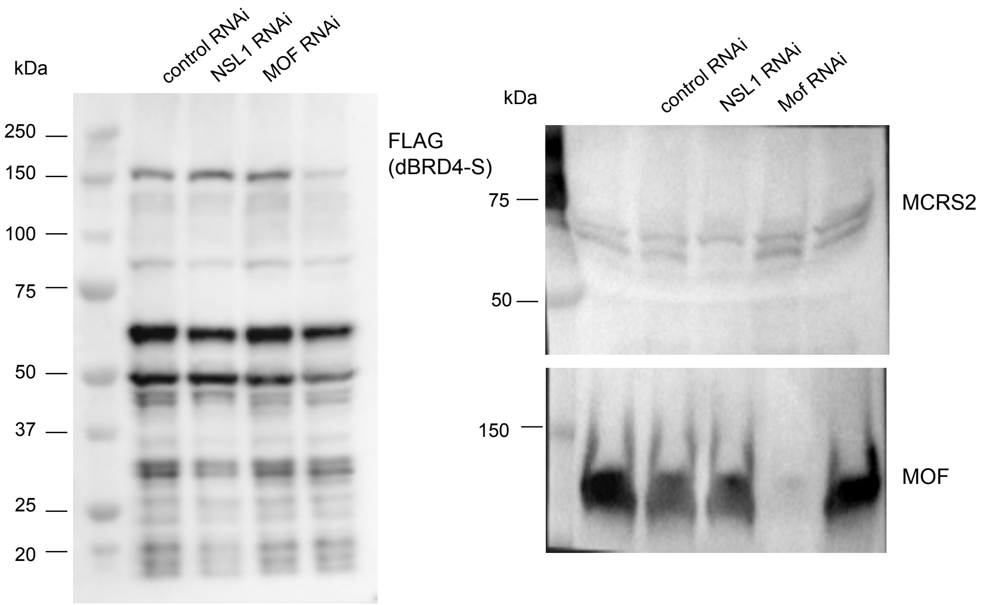

f. related to Supplementary Figure 5e

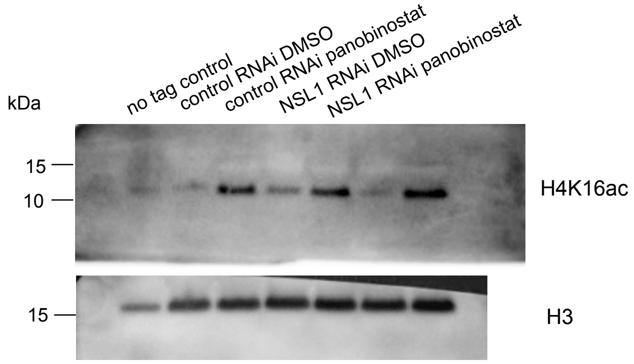

g. related to Supplementary Figure 6b

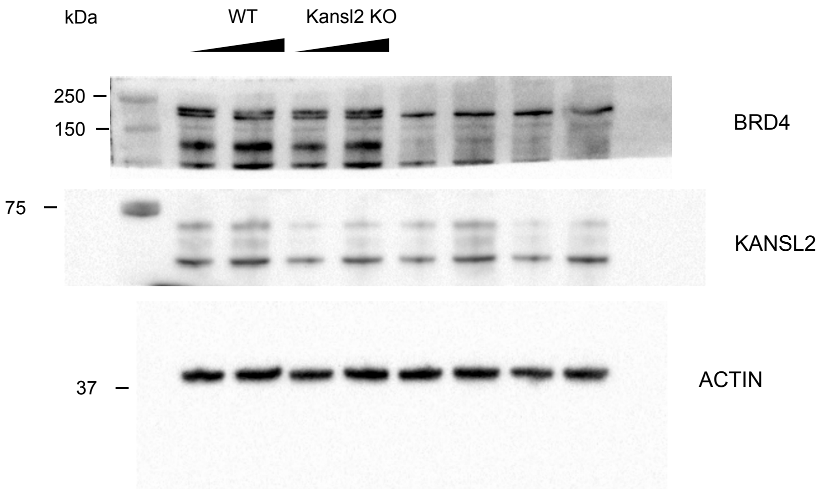

Supplementary Figure 8. Uncropped western blots

a.-g.) Uncropped western blots are shown, association to figures as indicated.

Supplementary References

1. Szklarczyk D*, et al.* STRING v11: protein–protein association networks with increased coverage, supporting functional discovery in genome-wide experimental datasets. *Nucleic Acids Res* **47**, D607-D613 (2018).

2. Kockmann T*, et al.* The BET protein FSH functionally interacts with ASH1 to orchestrate global gene activity in Drosophila. *Genome Biol* **14**, R18 (2013).

3. Chelmicki T*, et al.* MOF-associated complexes ensure stem cell identity and Xist repression. *Elife* **3**, e02024 (2014).

4. Gonzales-Cope M, Sidoli S, Bhanu NV, Won K-J, Garcia BA. Histone H4 acetylation and the epigenetic reader Brd4 are critical regulators of pluripotency in embryonic stem cells. *BMC Genomics* **17**, 95 (2016).

5. Bhagwat AS, Roe J-S, Mok BY, Hohmann AF, Shi J, Vakoc CR. BET bromodomain inhibition releases the mediator complex from select cis-regulatory elements. *Cell Rep* **15**, 519-530 (2016).

6. Winter GE*, et al.* Phthalimide conjugation as a strategy for in vivo target protein degradation. *Science* **348**, 1376-1381 (2015).
