# Supplementary material for: Evolutionary conserved NSL complex/BRD4 axis controls transcription activation via histone acetylation
Source: Nat Commun. 2020 May 7;11:2243. doi: 10.1038/s41467-020-16103-0 (PMC7206058; doi:10.1038/s41467-020-16103-0)
Supplement: Supplementary file 3 — Description of Additional Supplementary Information [file 41467_2020_16103_MOESM3_ESM.pdf]

Description of Additional Supplementary Files

File Name: Supplementary Data 1
Description: Scores of genome wide RNAi screen.

File Name: Supplementary Data 2
Description: List of differentially expressed (DE) genes obtained in RNA-seq from Drosophila S2 cells and human fibroblast cell lines.

File Name: Supplementary Data 3
Description: Table with primer sequences.
