# Supplementary material for: Evolutionary conserved NSL complex/BRD4 axis controls transcription activation via histone acetylation
Source: Nat Commun. 2020 May 7;11:2243. doi: 10.1038/s41467-020-16103-0 (PMC7206058; doi:10.1038/s41467-020-16103-0)
Supplement: Supplementary file 7 — Reporting Summary [file 41467_2020_16103_MOESM7_ESM.pdf]

natureresearch

Corresponding author(s): Akhtar Asifa

Last updated by author(s): Mar 25, 2020

Reporting Summary

Nature Research wishes to improve the reproducibility of the work that we publish. This form provides structure for consistency and transparency in reporting. For further information on Nature Research policies, see Authors & Referees and the Editorial Policy Checklist .

Statistics

For all statistical analyses, confirm that the following items are present in the figure legend, table legend, main text, or Methods section.

n/a	Confirmed	
☐	☒	The exact sample size (*n*) for each experimental group/condition, given as a discrete number and unit of measurement
☐	☒	A statement on whether measurements were taken from distinct samples or whether the same sample was measured repeatedly
☐	☒	The statistical test(s) used AND whether they are one- or two-sided<br>*Only common tests should be described solely by name; describe more complex techniques in the Methods section.*
☒	☐	A description of all covariates tested
☐	☒	A description of any assumptions or corrections, such as tests of normality and adjustment for multiple comparisons
☐	☒	A full description of the statistical parameters including central tendency (e.g. means) or other basic estimates (e.g. regression coefficient) AND variation (e.g. standard deviation) or associated estimates of uncertainty (e.g. confidence intervals)
☐	☒	For null hypothesis testing, the test statistic (e.g. *F*, *t*, *r*) with confidence intervals, effect sizes, degrees of freedom and *P* value noted<br>*Give P values as exact values whenever suitable.*
☒	☐	For Bayesian analysis, information on the choice of priors and Markov chain Monte Carlo settings
☒	☐	For hierarchical and complex designs, identification of the appropriate level for tests and full reporting of outcomes
☒	☐	Estimates of effect sizes (e.g. Cohen's *d*, Pearson's *r*), indicating how they were calculated

Our web collection on statistics for biologists contains articles on many of the points above.

Software and code

Policy information about availability of computer code

Data collection	See "ChIP-seq Data analysis and RNA-seq analysis" in the Manuscript Methods section for details.
Data analysis	RNA seq: For Drosophila samples, reads were mapped to the Drosophila genome (dm6) using subreads , transcripts were counted with htseq , differential expression analysis was performed using DESeq2 (v1.18.1), see Manuscript Methods section for details. Downstream analysis and visualization was done using R (v3.4.1.).<br>RNA seq from fibroblast cell lines was mapped to the human genome (hg38) using STAR, transcripts were counted using feature counts. Differential gene expression analysis was performed using DESeq2.<br>ChIP seq: Sequencing reads of ChIP samples and their respective input samples were trimmed using TrimGalore (developed by Felix Krueger, Babraham Institute), subsequently mapped to the Drosophila genome (dm6) using Bowtie2 . deepTools2 was used to generate Input normalized coverage files. Peaks were called using MACS2. Further details in Manuscript Methods section. Downstream analysis and visualization was done using R (v3.4.1.).<br>IPA (Ingenuity Pathway Analysis) v01-07 was used to generate Perturbation Pathway analysis.<br>Further analysis are stated in methods section in details

For manuscripts utilizing custom algorithms or software that are central to the research but not yet described in published literature, software must be made available to editors/reviewers. We strongly encourage code deposition in a community repository (e.g. GitHub). See the Nature Research guidelines for submitting code & software for further information.

Data

Policy information about availability of data

All manuscripts must include a data availability statement. This statement should provide the following information, where applicable:

- Accession codes, unique identifiers, or web links for publicly available datasets
- A list of figures that have associated raw data
- A description of any restrictions on data availability

RNA-seq and ChIP-seq data have been deposited to Gene Expression Omnibus (GEO) under the accession number: GSE135815 [https://www.ncbi.nlm.nih.gov/geo/query/acc.cgi?acc=GSE135815] and are also accessible under BioProject PRJNA560185 [https://www.ncbi.nlm.nih.gov/bioproject/PRJNA560185].
The source data underlying Figs 2a, 3b-d, 4d-g, 5b, 6a,b and Supplementary Figs 1a,b, 3a-c, 4b, 4g,h, 5f,g, 6c-e, 7a,b are provided as a Source Data file.

Field-specific reporting

Please select the one below that is the best fit for your research. If you are not sure, read the appropriate sections before making your selection.

☒ Life sciences ☐ Behavioural & social sciences ☐ Ecological, evolutionary & environmental sciences

For a reference copy of the document with all sections, see nature.com/documents/nr-reporting-summary-flat.pdf

Life sciences study design

All studies must disclose on these points even when the disclosure is negative.

Sample size	Sample size, number of replicates, error bars and statistical tests were chosen based on accepted practices in the field and stated in each figur e legend. Generally, experiments were performed independently and reproduced using at least three biological replicates. Exceptions for exceptionally rare/difficult to collect samples / genotypes are noted and justified in figure legends and methods.
Data exclusions	N/A
Replication	Each experiment was repeated multiple times with similar results, all attempts at replication were successful. Details on replication of each particular experiment are provided in Figure Legends.
Randomization	The manuscript contains individual samples from patients received from different countries.
Blinding	Investigators were not blinded. Blinding was not relevant as all read-outs were objective.

Reporting for specific materials, systems and methods

We require information from authors about some types of materials, experimental systems and methods used in many studies. Here, indicate whether each material, system or method listed is relevant to your study. If you are not sure if a list item applies to your research, read the appropriate section before selecting a response.

Materials & experimental systems

n/a	Involved in the study	
☐	☒	Antibodies
☐	☒	Eukaryotic cell lines
☒	☐	Palaeontology
☐	☒	Animals and other organisms
☐	☒	Human research participants
☒	☐	Clinical data

Methods

n/a	Involved in the study	
☐	☒	ChIP-seq
☒	☐	Flow cytometry
☒	☐	MRI-based neuroimaging

Antibodies

Antibodies used	dBRD4 (Paro lab, ID166), Pol2 ser2p (ab5095, Abcam), Pol2 ser5p (ab5131, Abcam), Rpb3 (Akhtar lab), H3 (ab10799, Abcam), NSL3 (Akhtar lab), MOF (Akhtar lab), H2A.V (61752, Active Motif), MCRS2 (Akhtar lab), H4K16ac (07-329, Millipore), Kansl2 (HPA038497, Sigma), BRD4(A301-985, Bethyl), Actin-HRP(sc-1616, Santa Cruz), GAPDH-HRP (MA5-15738, Thermo Scientific), FLAG-HRP(A8592, Sigma) and HA (901533, Biolegend).
Validation	Data are provided in the manuscript and catalog number from company is stated. Non-commercial antibodies have been validated in previously published papers. For ChIP-seq and ChIP-qPCR, mutants and untagged controls validate the specific enrichments, where possible.

Eukaryotic cell lines

Policy information about cell lines

Cell line source(s)	Drosophila S2 cells were purchased from Thermo Fisher (R690-07). Fibroblast cell lines and mouse embryonic stem cells have been established as described in the method section. Patient samples used in the study are restricted as they need consent from the patient families for further use.
Authentication	mESC have been tested for pluripotency markers (as in manuscript), alkaline phosphatase and colony formation assay. RNAseq from fibroblasts confirms their nature. S2 cells were confimed by multiple RNAseq experiments.
Mycoplasma contamination	The fibroblast cells were regularly tested for mycoplasma by PCR (Jena Biosciences PP-401)
Commonly misidentified lines (See ICLAC register)	N/A

Animals and other organisms

Policy information about studies involving animals; ARRIVE guidelines recommended for reporting animal research

Laboratory animals	mus musculus, following strains were crossed for generating mouse embryoinic stem cells: tm1a(EUCOMM)Wtsi, purchased from (IKMC) and CAG::CreERT2, purchased from Jackson laboratory.
Wild animals	*Provide details on animals observed in or captured in the field; report species, sex and age where possible. Describe how animals were caught and transported and what happened to captive animals after the study (if killed, explain why and describe method; if released, say where and when) OR state that the study did not involve wild animals.*
Field-collected samples	*For laboratory work with field-collected samples, describe all relevant parameters such as housing, maintenance, temperature, photoperiod and end-of-experiment protocol OR state that the study did not involve samples collected from the field.*
Ethics oversight	Animal husbandry and all experiments were approved by the committee on ethics of animal experiments of the state Baden-Württemberg (Regierungspräsidium Freiburg).

Note that full information on the approval of the study protocol must also be provided in the manuscript.

Human research participants

Policy information about studies involving human research participants

Population characteristics	Patients were recruited in the pediatric clinic when KANSL1 mutation was identified.
Recruitment	The individuals screened in this study had all been referred to our research program with a diagnosis of KANSL1 variant. The affected individual and/or their family gave written consent for their inclusion in the analysis under a local ethic committees
Ethics oversight	Ethics approval (S60206) to Leuven University for control samples and (DC-2008-735) to Université Grenoble-Alpes CHU for patient samples.

Note that full information on the approval of the study protocol must also be provided in the manuscript.

ChIP-seq

Data deposition

☒ Confirm that both raw and final processed data have been deposited in a public database such as GEO.

☒ Confirm that you have deposited or provided access to graph files (e.g. BED files) for the called peaks.

Data access links<br>*May remain private before publication.*	GEO accession GSE135815<br>Data access link: https://www.ncbi.nlm.nih.gov/geo/query/acc.cgi?acc=GSE135815
Files in database submission	D.melanogaster ChIPseq, D.melanogaster RNAseq, patient fibroblast RNAseq
Genome browser session (e.g. UCSC)	*Provide a link to an anonymized genome browser session for "Initial submission" and "Revised version" documents only, to enable peer review. Write "no longer applicable" for "Final submission" documents.*

Methodology

Replicates	at least two biological replicates were used. Exact numbers are mentioned in respective figure legends
Sequencing depth	minimum 10mio reads per sample

Antibodies	H4K16ac (Millipore), H3 (ab10799, Abcam), dBRD4 (Paro lab, ID166) or Rbp3 (Akhtar lab) antibody 4ul for 10ug chromatin in S2 cells, Streptavidin (65601, Thermo Fisher)
Peak calling parameters	peaks of endogenous dBRD4 ChIP and dBRD4-S biotin ChIP were called using MACS2 with the following parameters: band width, 300; model fold, 5 to 50; P value cut-off, 5x 10-5; if a called peak region overlapped with TSS +/- 200bp the gene was defined as bound gene.
Data quality	Sequencing reads of ChIP samples and their respective input samples were trimmed using TrimGalore using a quality threshold of 20. Data quality and enrichment was ensured with biological controls (i.e. untagged samples)
Software	No custom codes were used. Published scripts and packages used to analyze data are described in Materials & Methods.
